# Comparing the psychometric properties of two primary school Computational Thinking (CT) assessments for grades 3 and 4: The Beginners' CT test (BCTt) and the competent CT test (cCTt)

**DOI:** 10.3389/fpsyg.2022.1082659

**Published:** 2022-12-12

**Authors:** Laila El-Hamamsy, María Zapata-Cáceres, Pedro Marcelino, Barbara Bruno, Jessica Dehler Zufferey, Estefanía Martín-Barroso, Marcos Román-González

**Affiliations:** ^1^MOBOTS Group, Ecole Polytechnique Fédérale de Lausanne, Lausanne, Switzerland; ^2^LEARN - Center for Learning Sciences, Ecole Polytechnique Fédérale de Lausanne, Lausanne, Switzerland; ^3^Laboratory of Information Technologies in Education, Rey Juan Carlos University, Madrid, Spain; ^4^Computational Thinking Department, TreeTree2 (T2), Lisbon, Portugal; ^5^CHILI Laboratory, Ecole Polytechnique Fédérale de Lausanne, Lausanne, Switzerland; ^6^Faculty of Education, Universidad Nacional de Educación a Distancia (UNED), Madrid, Spain

**Keywords:** Computational Thinking, assessment, primary school, validation, developmental appropriateness, psychometrics

## Abstract

**Introduction:**

With the increasing amount of research around Computational Thinking (CT) and endeavors introducing CT into curricula worldwide, assessing CT at all levels of formal education is of utmost importance to ensure that CT-related learning objectives are met. This has contributed to a progressive increase in the number of validated and reliable CT assessments for K-12, including primary school. Researchers and practitioners are thus required to choose among multiple instruments, often overlapping in their age validity.

**Methods:**

In this study, we compare the psychometric properties of two of these instruments: the Beginners' CT test (BCTt), developed for grades 1–6, and the competent CT test (cCTt), validated for grades 3–4. Classical Test Theory and Item Response Theory (IRT) were employed on data acquired from 575 students in grades 3–4 to compare the properties of the two instruments and refine the limits of their validity.

**Results:**

The findings (i) establish the detailed psychometric properties of the BCTt in grades 3–4 for the first time, and (ii) through a comparison with students from the same country, indicate that the cCTt should be preferred for grades 3–4 as the cCTt is able to discriminate between students of low and medium ability. Conversely, while the BCTt, which is easier, shows a ceiling effect, it is better suited to discriminate between students in the low ability range. For these grades, the BCTt can thus be employed as a screening mechanism to identify low ability students.

**Discussion:**

In addition to providing recomendations for use of these instruments, the findings highlight the importance of comparing the psychometric properties of existing assessments, so that researchers and practitioners, including teachers and policy makers involved in digital education curricular reforms, may take informed decisions when selecting assessments.

## 1. Introduction and related work

Computational Thinking (CT) is more and more often considered to be an essential twenty-first century skill (Li et al., 2020), that is as important as reading, writing, and arithmetic (Wing, 2006) and must be taught at a young age. Despite the lack of consensus regarding the definition of CT, CT is traditionally defined by Wing (2006) as “an approach to solving problems, designing systems, and understanding human behavior that draws on concepts fundamental to computing” which was later reformulated by Aho (2012) as “the thought processes involved in formulating problems so their solutions can be represented as computational steps and algorithms.” As such CT has often been associated with Computer Science (CS), although many researchers consider CT to be transversal (Mannila et al., 2014; Weintrop, 2016; Denning and Tedre, 2021; Weintrop et al., 2021b), and not exclusively related to CS or mathematics (Li et al., 2020). This has lead to a “tremendous growth in curricula, learning environments, and innovations around CT education” (Weintrop et al., 2021b). To be successful, these initiatives rely on the constructive alignment between the learning objectives, teaching and learning activities, and *assessments* (Biggs, 1996). Developing and implementing effective CT interventions thus requires expanding the portfolio of developmentally appropriate instruments to assess CT at all levels of formal education, for use by researchers and educators alike (Weintrop et al., 2021a).

Developing CT assessments requires having better insight into what composes this competence, with a competence referring to “the proven ability to use knowledge, skills, and personal, social, and/or methodological abilities, in work or study situations and in professional and personal development” (European Union, 2006). As such, Brennan and Resnick (2012) proposed an operational definition of CT by decomposing CT into three dimensions. The first is CT-concepts, i.e., “the concepts designers engage with as they program, such as iteration, parallelism,” (Brennan and Resnick, 2012), which thus includes sequences, loops, if-else statements and so forth at the primary school level. These elements can be adequately assessed through diagnostic and summative tools (Román-González et al., 2019). The second is CT-practices i.e., “the practices designers develop as they engage with the concepts, such as debugging projects or remixing others' (Brennan and Resnick, 2012), which thus requires understanding the thought processes involved in resolving CT problems. These may include elements of abstraction, decomposition, evaluation, and so forth and can be adequately assessed through formative-iterative tools and data-mining tools (Román-González et al., 2019). The third is CT-perspectives, i.e., “the perspectives designers form about the world around them and about themselves” (Brennan and Resnick, 2012), and therefore their perception of CT which can be adequately evaluated through perception and attitude scales and vocabulary assessments (Román-González et al., 2019).

Despite the increase in research around CT in the past two decades, and the various means of assessing CT identified by Tang et al. (2020) [i.e., “traditional test(s) composed of selected- or constructed response questions, portfolio assessment(s), interviews, and surveys”], few validated and reliable instruments exist for CT, and even less at the primary school level (Román-González et al., 2019; Basu et al., 2020; Zapata-Cáceres et al., 2020; Clarke-Midura et al., 2021). This limitation was highlighted by Tang et al. (2020) in their recent meta review on CT assessments: out of 96 studies, only 45% provided reliability evidence and just 18% provided validity evidence. This mirrors the findings of Bakala et al. (2021) who, in their literature review on the effects of robots on preschool children's CT, found that most studies employed *ad-hoc* evaluations, typically neither standardized nor validated. Bakala et al. (2021) attributed this to the fact that only two recent valid and reliable tests for that age group existed at the time of their review [the TechCheck by Relkin et al., 2020; Relkin and Bers, 2021 and the Beginners' CT test (BCTt) by Zapata-Cáceres et al., 2020] and recommended that researchers aim to employ them in future studies. To further limit the available choices, many existing assessments are strongly tied to specific CS frameworks (Rowe et al., 2021) [e.g., Dr., Scratch (Moreno-León and Robles, 2015) or the Fairy assessments (Werner et al., 2012)]. As stated by Relkin and Bers (2021) and Rowe et al. (2021), being strongly tied to specific frameworks means that the instrument risks conflating with programming abilities. This contributes to a lack of generalizability and thus limits the range of applications of such instruments (Tikva and Tambouris, 2021), which for example should be avoided in the context of pre-post test experimental designs. It is essential to provide researchers and practitioners (e.g., teachers and policy makers involved in digital education curricular reforms) the means to assess CT:

1. at all levels of education
2. independently from specific studies or programming environments
3. in a valid and reliable way to ensure that there is sufficient “evidence and theory [to] support the interpretations of test scores entailed by proposed uses of tests” (Clarke-Midura et al., 2021)
4. with an instrument which can easily be administered.

Without these, it is not possible to ensure that CT-related learning objectives are met, whether in individual interventions or in the context of large scale CS and/or CT curricular reform initiatives (El-Hamamsy et al., 2021a,b).

Unfortunately, while an increasing number of instruments have been recently developed, several do not meet these criteria (Hubwieser and Mühling, 2014; Bellettini et al., 2015; Gane et al., 2021; Parker et al., 2021). For example, the Bebras challenge is sometimes used to assess CT skills, but has undergone limited psychometric validation (Hubwieser and Mühling, 2014; Bellettini et al., 2015). Gane et al. (2021)'s assessment require manual grading and multiple annotators, thus limiting the test's scalability and its usability by other researchers and practitioners. Parker et al. (2021) assessment which is based on a combination of block-based and Bebras-style questions, has been piloted with just 57 fourth graders. Finally Chen et al. (2017)'s assessment for 5th graders appears highly dependent on the robotics programming context, includes open questions and was administered to just 37 students, thus including the limitations of all the aforementioned assessments, in addition to limiting its use in other CT-related contexts.

Instruments meeting the aforementioned criteria, and having undergone a psychometric validation and reliability assessment process at the level of primary school (see section 2.2), include the TechCheck for lower primary school (grades 1–2, ages 6–8, Relkin et al., 2020), the TechCheck-K, which is an adaptation of the former for kindergarden (ages 4–6, Relkin and Bers, 2021), the BCTt for grades 1–6 (ages 5–10, Zapata-Cáceres et al., 2020), the competent CT test (cCTt) for grades 3–4 (ages 7–9, El-Hamamsy et al., 2022a), the Computational Thinking Assessment for Chinese Elementary Students (CTA-CES) for grades 3–6 (ages 9–12, Li et al., 2021), and Kong and Lai (2022)'s CT-concepts test for grades 3–5. A synthesis of these instruments is provided in Table 1 and shows that these instruments often differ in the underlying definition of CT employed to define the test items which makes it complex to compare them pyschometrically. Furthermore, these instruments are all relatively new and adopt an unplugged approach, using multiple choice questions to assess primary school students' CT abilities. Furthermore, there is an overlap in their target age ranges. It is thus important for researchers and practitioners to not only identify instruments that best assess the learning objectives of their interventions, but also to understand the limits of validity of these instruments to make informed decisions for their own studies. Such instruments are unfortunately not often compared against one another to determine which may be more appropriate for a given age range. To the best of our knowledge, only the TechCheck and TechCheck-K were compared to establish whether the TechCheck-K would be an adequate instrument for kindergarden students (Relkin and Bers, 2021), with the TechCheck being more appropriate for first and second graders.

**Table 1:** Synthesis of validated and scalable primary school unplugged CT assessments and corresponding validation processes adapted from El-Hamamsy et al. (2022a).

**Test**	**Format**	**Target age group**	**CT definition**	**Validation process**	**Sample**	**Validity established for**
TechCheck (Relkin et al., 2020) and TechCheck-K (Relkin and Bers, 2021)	15 item MCQ	1st and 2nd graders (6–9 year old students) and kindergarden (5–6 year old students)	Algorithms, Modularity, Design Process, Debugging, Control Structures, Hardware/Software	Expert validation, psychometric analysis (Classical Test Theory and Item Response Theory), convergent validation with the TACTIC-KIBO	768 5–9 year old students participating in a robotics coding curriculum and 89 kindergarden students without coding experience	Full sample
Beginner's CT test (Zapata-Cáceres et al., 2020; Zapata-Cáceres and Fanchamps, 2021)	25 item MCQ	Primary school (5–12 year old students) and Kindergarden (4–5 years old students)	Computational concepts, practices, perspectives (Brennan and Resnick, 2012)	Expert validation, and psychometric analysis (Classical Test Theory)	299 primary school students from grades 1 to 6 and 5 kindergarden students	4–7 year old students
The competent CT test (cCTt) (El-Hamamsy et al., 2022a)	25 item MCQ	Primary school (7–9 year old students)	Computational concepts, practices, perspectives (Brennan and Resnick, 2012)	Expert validation and psychometric analysis (Classical Test Theory, Item Response Theory), Confirmatory Factor Analysis	1,519 primary school students from grades 3 to 4	Full sample
CT Assessment for Chinese Elementary Students (CTA-CES, Li et al., 2021)	25 item MCQ	Grades 3–6 (ages 9–12)	Abstraction, algorithmic thinking, decomposition, evaluation, pattern recognition, generalization (Selby and Woollard, 2013)	Expert validation, Classical Test Theory, Item Response Theory, Construct validity by comparing two groups of students, criterion validity through correlations with reasoning, spatial ability, and verbal ability	280 grade 3–6 students	Full sample
Kong and Lai (2022)'s CT-concepts test	14 item MCQ	Grades 3–5 (ages 8–10)	Sequences, conditionals, repetition (Brennan and Resnick, 2012)	Item Response Theory	13,670 grade 3 to 5 students	Full sample

In this paper, we are interested in the overlap between the BCTt and the cCTt for students in grades 3 and 4 as these two instruments overlap in their targets, and are from the same “family” of CT tests, and thus cover the same concepts. Therefore, the BCTt and cCTt cannot be considered complementary within a system of assessments, and thus require choosing between them. It is therefore essential to establish their limits of validity for the considered age group to provide recommendations to help researchers make an informed decision when selecting CT-assessments in accordance with their study requirements. Indeed, while the BCTt was initially developed as an instrument looking to cover all of primary school, the validation procedure appeared to indicate that the BCTt was too easy for students in upper primary school (Zapata-Cáceres et al., 2020). As the cCTt was derived from the BCTt to adapt the instrument in terms of format and content to improve its validity for students in grades 3 and 4 (7–9 year old students), the present study therefore investigates how the BCTt and cCTt complement each other in assessing CT in grades 3 and 4, to propose recommendations for their use for these grades. More specifically, we look to answer the following research questions:

1. *How do the psychometric properties of the BCTt and the cCTt compare for students in grades 3–4 (7–9 years old)?*
2. *How does the psychometric comparison inform us about how the instruments should be used in grades 3–4 (7–9 years old)?*

## 2. Methodology

### 2.1. The BCTt, cCTt, and their validation

**Figure 1:**
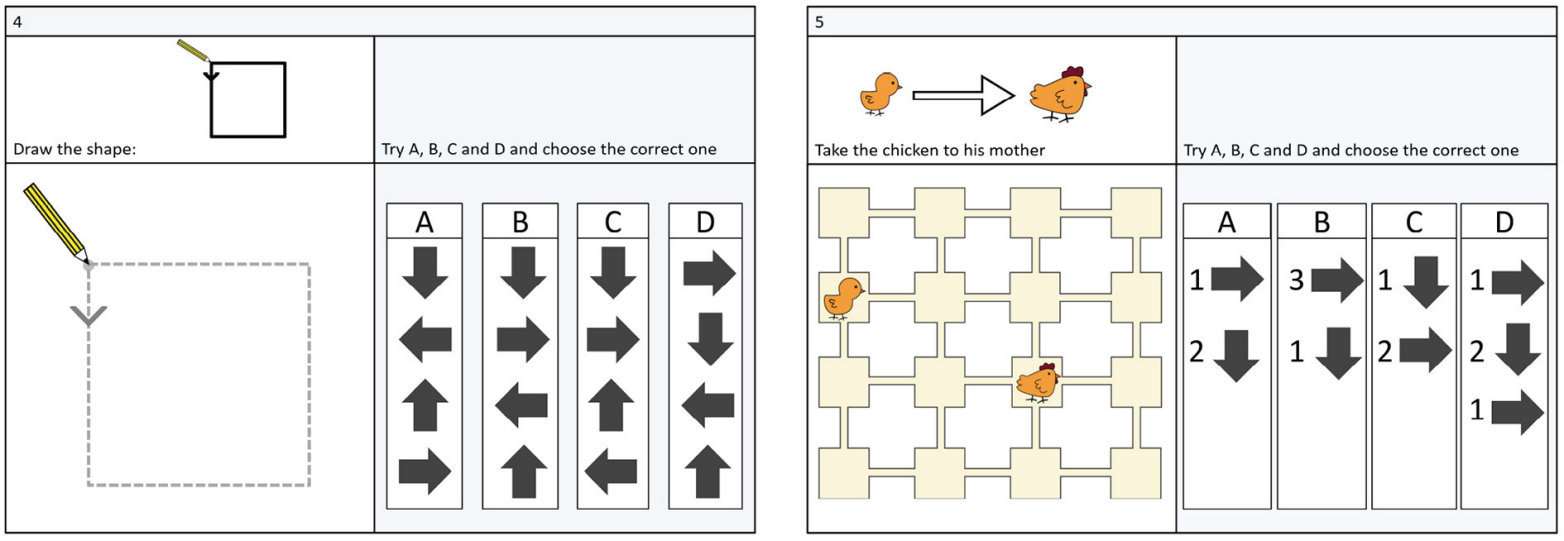
The two main question formats of the BCTt and cCTt: grid **(Left)** and canvas **(Right)** (Figure taken from El-Hamamsy et al., 2022a).

The BCTt and the cCTt are two 25-item multiple choice CT assessments^1^ of progressive difficulty, targeting the CT-concepts posed by Brennan and Resnick (2012) in their decomposition of CT into concepts, practices, and perspectives. More specifically, the two tests evaluate notions of sequences, simple loops (only one instruction is repeated), complex loops (two or more instructions are repeated), conditionals and while statements (see the distribution of items in Table 2), with the factor structure pertaining to these concepts having been validated through Confirmatory Factor Analysis by El-Hamamsy et al. (2022a). The BCTt was derived from the CTt (Román-González et al., 2017, 2018, 2019), with changes in terms of format and content to adapt it to primary school. In a similar spirit, the cCTt made alterations to the format and content of the BCTt to more specifically target students in grades 3 and 4 (El-Hamamsy et al., 2022a). Both instruments, like their predecessor the CTt, employ grid-type and canvas-type questions (see Figure 1) and employ the same type of tasks. The individual questions differ (see Table 2) as the cCTt (i) favors questions on 4 × 4 grids, (ii) replaces BCTt questions of low difficulty with questions related to complex concepts (e.g., while statements), (iii) alters the disposition of objects on the grids, and responses, with respect to the BCTt equivalents.

**Table 2:** Comparison between the BCTt and the cCTt in terms of question concepts and question types (Table taken from El-Hamamsy et al., 2022a).

		**BCTt**				**cCTt**		
**Blocks**	**Grid** (3 × 3)	**Grid** (4 × 4)	**Canvas**	**Total**	**Grid** (3 × 3)	**Grid** (4 × 4)	**Canvas**	**Total**
Sequences	3	1	2	6	1	1	2	4
Simple loops	3	2	0	5	0	4	0	4
Complex loops	0	5	2	7	0	5	2	7
Conditional statements	1	3	0	4	1	3	0	4
While statements	1	2	0	3	1	3	0	4
Combinations	0	0	0	0	0	2	0	2
Total	8	13	4	25	3	18	4	25

Both the BCTt and cCTt instruments were validated by starting with an evaluation by experts and making adjustments based on their suggestions, prior to administration to students in the target age groups. The BCTt, which was designed for grades 1–6, was administered to 200 students in that age group (Zapata-Cáceres et al., 2020). The authors found that the test had good reliablity with Cronbach's α = 0.824 . The results indicated that the students improved as they got older, and started to exhibit a ceiling effect in grades 3–4^2^. The results indicated that the differences were significant between all grades, excepted those in grades 4–6 who already exhibit a ceiling effect (Zapata-Cáceres et al., 2020). These results indicate that students begin to exhibit a ceiling effect either in grade 3 or grade 4. The cCTt, which was designed for grades 3 and 4, was administered to 1,519 students in that age group and analyzed through Classical Test Theory and Item Response Theory (El-Hamamsy et al., 2022a). The results indicated that the grade 4 students scored significantly better than the grade 3 students (out of 25 pts, the one-way ANOVA indicates that *p* < 0.001 , Δ_*grades*_ = +2.9 pts, Cohen's *d* = 0.57 , μ_3_ = 12.62 ± 5.18 , *n* = 711; μ_4_ = 15.49 ± 4.96 , *n* = 749). The Classical Test Theory results indicated that the test had good reliability with Cronbach's α = 0.85 , levels of discrimination, and a wide range of question difficulties. Item Response Theory was employed to support these findings and indicated that the test was better suited at evaluating and discrimination between students with low and medium abilities.

### 2.2. Psychometric analysis

The objective of this study is to compare the psychometric properties of the BCTt and cCTt for students in grades 3 and 4. Classical Test Theory and Item Response Theory are two complementary (De Champlain, 2010; Awopeju and Afolabi, 2016) approaches typically employed to analyse the validity and reliability of scales and assessments. The Classical Test Theory and Item Response Theory (IRT) analyses are conducted in R (version 4.2.1, R Core Team, 2019) using the following packages: lavaan (version 0.6-11, Rosseel, 2012), CTT (version 2.3.3, Willse, 2018), psych (version 2.1.3, Revelle, 2021), mirt (version 1.36.1, Chalmers, 2012), and subscore (version 3.3, Dai et al., 2022).

#### 2.2.1. Classical test theory

Classical Test Theory “comprises a set of principles that allow us to determine how successful our proxy indicators are at estimating the unobservable variables of interest” (DeVellis, 2006). Classical test theory focuses on test scores (Hambleton and Jones, 1993) and computes:

- Reliability of the scale using Cronbach's α measurement of internal consistency of scales (Bland and Altman, 1997). In the context of assessments, 0.7 < α < 0.9 is considered high and 0.5 < α < 0.7 is considered moderate (Hinton et al., 2014; Taherdoost, 2016). The drop alpha is computed per question as it indicates of the reliability of the test without said question, and thus whether the internal consistency of the test improves without it.
- Item difficulty index, i.e., the proportion of correct responses. *Please note that this means that a question with a high difficulty index is an easy question*. Determining whether questions are too easy or too difficult is often based on arbitrary thresholds which vary around what are considered to be ideal item difficulties. Indeed, some researchers have posited that item difficulties should vary between 0.4 and 0.6 as these are claimed to have maximum discrimination indices (Vincent and Shanmugam, 2020). As such, thresholds employed in the literature have varied around these values, with items being classified as difficult for a range of thresholds between 0.1 and 0.3, and items being classified as easy for a range of thresholds varying between 0.7 and 0.9.
  In this study, to remain coherent with the first cCTt validation in grades 3–4, we consider that questions with a difficulty index above 0.85 are too easy, while those with a difficulty index below 0.25 are too hard and could be revised.
- Point biserial correlation, or item discrimination. This is a measure of discrimination between the high ability examinees and low ability examinees. A point-biserial correlation above 0.15 is recommended, with good items generally having point biserial correlations above 0.25 (Varma, 2006). In this article, we consider a threshold of 0.2 , which is commonly employed in the field (Chae et al., 2019).

Unfortunately, Classical Test Theory suffers from several limitations, including that the analysis is sample-dependent (Hambleton and Jones, 1993). As such, analyzing an instrument from the lens of Classical Test Theory on two different populations may not yield consistent results. The literature thus recommends employing Item Response Theory to complement the results of Classical Test Theory.

#### 2.2.2. Item Response Theory (IRT)

According to Hambleton and Jones (1993), (i) IRT *is sample independent* so scores describing examinee proficiency are not dependent on the test difficulty, (ii) test items can be matched to ability levels, and (iii) the test models do not require strict parallel tests to assess reliability. This is because IRT models the link between a students' latent ability and their probability of correctly answering a question. Indeed, by evaluating the tests' questions with respect to latent ability:

- The results are more likely to be sample independent, and therefore more likely to generalize beyond a specific sample of learners (Xie et al., 2019), thus providing consistency between two different populations.
- Item Response Theory is more adapted to compare multiple assessments through the latent ability scale (Jabrayilov et al., 2016; Dai et al., 2020), and thus including cases where different populations have taken the tests. Comparing two assessments can indeed be done in cases where the instruments measure the same latent traits (Xie et al., 2019), which we believe is possible in the present case because both instruments measure the same CT-concepts, using the same symbols. This can be verified through Confirmatory Factor Analysis, as done by Kong and Lai (2022).

**Figure 2:**
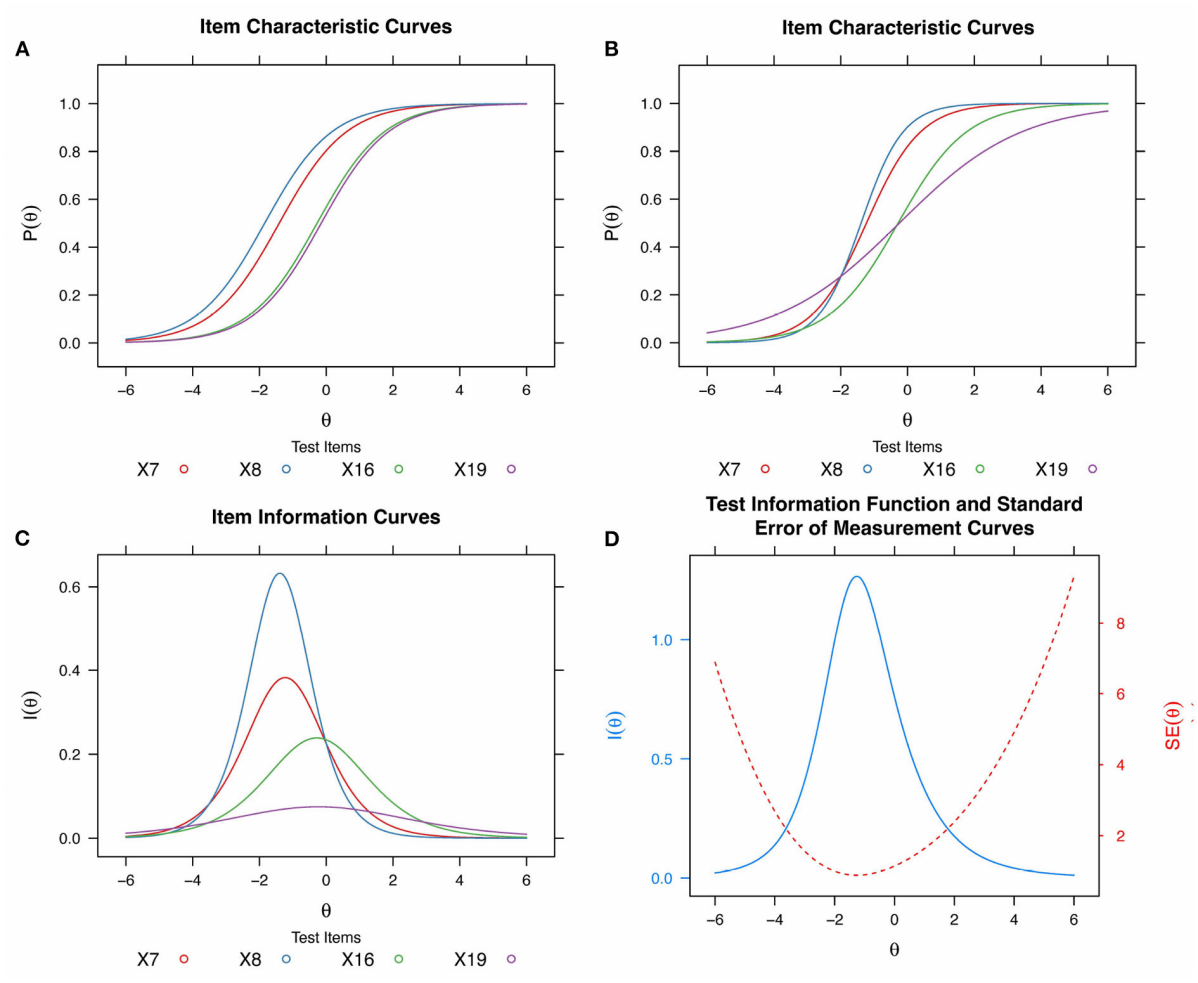
IRT Theory plots. **(A)** Item Characteristic Curves for four items of equal discrimination (slope) and varying difficulty (using a 1-PL model on the cCTt test data). **(B)** Item Characteristic Curves (ICC) for four items (blue, red, green, purple) of varying difficulty and discrimination (using a 2-PL model on cCTt test data). **(C)** Item Information Curves (IICs) for the items in **(B)**. **(D)** Test Information Function (TIF, in blue) for the four items from Panels **(B)** and **(C)** (IIC, in black), and the standard error of measurement (SEM, in red).

Item Response Theory models estimate the probability of a person of a given ability (measured in standard deviations from the mean) answering each question correctly. This is visualized through a logistic Item Characteristic Curve (ICC) for each question. As Figure 2A shows, an item's difficulty (*b*_*i*_) is the *x*-value (θ) where the ICC reaches a *y* = 0.5 probability of answering correctly, and represents the number of standard deviations from the mean the question difficulty is. Items to the left of the graph are considered easier while items on the right are considered harder. According to De Ayala and Little (2022), “typical item and person locations fall within -3 to +3”, with easy items having scores below -2, average items having scores between -2 and +2 and hard items having scores above +2.

Several IRT models exist for binary response data, however given the low sample size (Sahin and Anil, 2017), we focus on one parameter logistic (1-PL) and 2-PL models. While 1-PL models consider that only difficulty varies across items, 2-PL models also take into account that some questions can discriminate more or less well between students of different ability, and thus exhibit varying ICC slopes. In the example in Figure 2B, blue and red items are of equal difficulty *b*_*i*_ (*y* = 0.5 crossing) and relatively similar discrimination *a*_*i*_ , while items green and purple are of equal difficulty and varying discrimination. As the blue item is steeper, it has a higher discrimination than the black and green items. According to De Ayala and Little (2022), reasonably good discrimination values range from approximately 0.8–2.5. Indeed, questions with steeper ICC slopes are better suited at discriminating between students at a given ability, while questions with lower discrimination power have more gentle slopes.

Items that discriminate better (steeper ICC slopes) thus provide more information about the ability level at which students are likely to start answering correctly, which results in higher bell shaped Item Information Curves, or IICs. The bell shaped curves in Figure 2C represent the amount of information *I*_*i*_ provided for each of the test's items according to the student's ability θ . These IICs vary in both maximum value (dependent on the item's discriminability, i.e., the ICC slope), and the *x*-value at which they reach it (the item's difficulty). Here, the blue and red curves, as well as the green and purple curves, have the same difficulty (they both reach their maximum around *x* = -2 and *x* = 0, respectively), but are of different discriminability: the blue item discriminates more than the red, the red more than the green and the green more than the purple (steeper ICC slope, and higher maximum IIC value).

Taking into account the different test items and the amount of information provided by each question, one can obtain the resulting Test Information Function (TIF) and Standard Error of Measurements (SEM). In Figure 2D, the TIF (blue) is the sum of the instrument's IICs from Figures 2B,C, while the SEM is the square root of the variance. The TIF shows that the instrument displays maximum information around -2 and provides more information in the low-medium ability range than in the high ability range. The SEM (red) is at its lowest where the test provides the most information (maximum of the TIF) and at its highest where the test provides the least information (minimum of the TIF).

Please note that prior to applying IRT, it is recommended to verify whether the data meets the unidimensionality criteria. If the unidimensionality criteria is not met, the higher the misspecification, then the higher the impact on the estimated parameters, and in particular on the discriminatoin parameter (with little impact on the difficulty parameter, Kahraman, 2013; Rajlic, 2019). The unidimensionality criteria can be verified through Confirmatory Factor Analysis (CFA) as done by Kong and Lai (2022) for instance. As the input data is binary (with a score of 0 or 1 per question), the CFA analysis is conducted using an estimator which is adapted to non-normal data and employs diagonally weighted least squared and robust estimators to estimate the model parameters (Schweizer et al., 2015; Rosseel, 2020).

When analyzing the results of IRT, as in the case of Confirmatory Factor Analysis, and other similar statistical approaches, multiple fit indices should be considered to establish the goodness of fit of the model. Model fit indices include the following metrics:

- The chi-square χ^2^ statistic which should have $p_{\chi^{2}}>0.05$ . However, the larger the sample, the larger the χ^2^ statistic, and the lower the *p*-value (Prudon, 2015; Alavi et al., 2020). The literature therefore suggests employing the ratio between the χ^2^ statistic and the degrees of freedom with a cutoff at χ^2^/*df* ≤ 3 (Kyriazos, 2018). At the individual item level for IRT models, Orlando and Thissen's signed χ^2^ statistic (*S*−χ^2^ ) is recommended, with a ratio of χ^2^/*df* ≤ 5 being acceptable (Wheaton et al., 1977; Kong and Lai, 2022) and a ratio below 3 being considered good.
- The root mean square error of approximation or RMSEA which should be < 0.06 for good fit and < 0.08 for acceptable fit (Hu and Bentler, 1999; Chen et al., 2008; Xia and Yang, 2019).
- The standardized root mean square residual or SRMR (Hu and Bentler, 1999; Xia and Yang, 2019) which should be < 0.08 .
- The comparative fit index (CFI) and Tucker Lewis index (TLI) with values >0.95 indicating a good fit, and acceptable values being >0.90 (Kong and Lai, 2022).

Finally, more specifically to IRT, are

- Yen (1984)'s Q3 statistic to measure local independence which requires that none of the pairs of item residuals have a high correlation to ensure that local independence is not violated for the given model type. Critical values for the Q3 statistic are often arbitrary (Christensen et al., 2017) (e.g., 0.2 Christensen et al., 2017; Kong and Lai, 2022 or 0.3 Marais, 2012). As in our case the sample size is small (around 200 for the cCTt and 300 for the BCTt), and the number of items is high, the threshold of 0.3 is chosen as a critical value as the Q3 statistic is expected to be higher here than in cases with large samples and low number of items (Christensen et al., 2017). Similarly, as the number of items is high, the critical values are also expected to be higher (Christensen et al., 2017). As such, we consider the 0.3 threshold for the present study.
  The Q3 statistic is computed once the model with the best fit has been selected.
- The *M*_2_ statistics by Maydeu-Olivares and Joe “which have been found to be effective in evaluating the goodness of fit of IRT models” (Kong and Lai, 2022).
- The IRT reliability for each ability θ which is “closely related to test information and standard error, as it concerns the measurement precision and can be calculated with the equation *r* = 1−*SEM*(θ^2^)” (Kong and Lai, 2022) where SEM represents the SEM for each ability.
- Wainer and Thissen (2001)'s marginal reliability metric (*r*_*xx*_) which “denotes the ratio of the true score variance to the total variance, expressed with respect to the estimated latent abilities” (Andersson and Xin, 2018).

### 2.3. Participants and data collection

To compare the instruments, we used data collected by researchers and practitioners using the BCTt and cCTt in a study looking to evaluate the impact of a CT intervention conducted in public schools in Portugal. The recruitment for the intervention was done in three stages. First a call was sent out to schools and teachers to ask whether they were interested in participating in the CT intervention which included a pre-post test assessment using either the BCTt (in spring 2020) or the cCTt (in spring 2021). Secondly, teachers who were interested were briefed about the intervention and the assessments before agreeing or not to participate with their classrooms. Thirdly, consent forms were sent out to the parents of the concerned students.

The administration of both instruments was done in the classrooms following the protocol established for the BCTt, and its adaptation for the cCTt. In order to compare the instruments and avoid biases from the interventions themselves (whose goals and outcomes are outside the scope of this article), we only consider the results of the pre-tests administered to 575 students prior to the interventions (El-Hamamsy et al., 2022b).^3^ More specifically, we analyse the results of the BCTt pre-test administered in March 2020 to 374 students in grades 3–4, and the results of the cCTt pre-test administered in April 2021 to 201 other students in grades 3–4 (see Table 3). All participants were enrolled in the same school districts in Portugal and did not have any prior experience with the CT-concepts measured with the instruments, as this is not part of the national curriculum. Please note that while the populations are not identical, they are considered to be comparable, and a comparison of both instruments is possible through the lens of IRT which is sample agnostic (see section 2.2.2) and complements the results of Classical Test Theory which may be subject to sample dependency. Comparing the properties of the instruments on two distinct samples also helps avoid the testing-effect, i.e., having students' performance improve on the second instrument because the questions employ the same modalities as the first instrument, and are therefore familiar and easier due to practice, rather than being due to a difference between the instruments (Knapp, 2016).

**Table 3:** Participants.

**Number of participants per grade**					
**Test**	**Gender**	**Grade 3**	**Grade 4**	**Undisclosed**	**Total**
BCTt	Female	80	82	5	167
	Male	78	61	6	145
	Undisclosed			62	62
	Total	158	143	73	374
cCTt	Female	36	68		104
	Male	38	59		97
	Total	74	127		201

## 3. Results

### 3.1. Score distribution

**Figure 3:**
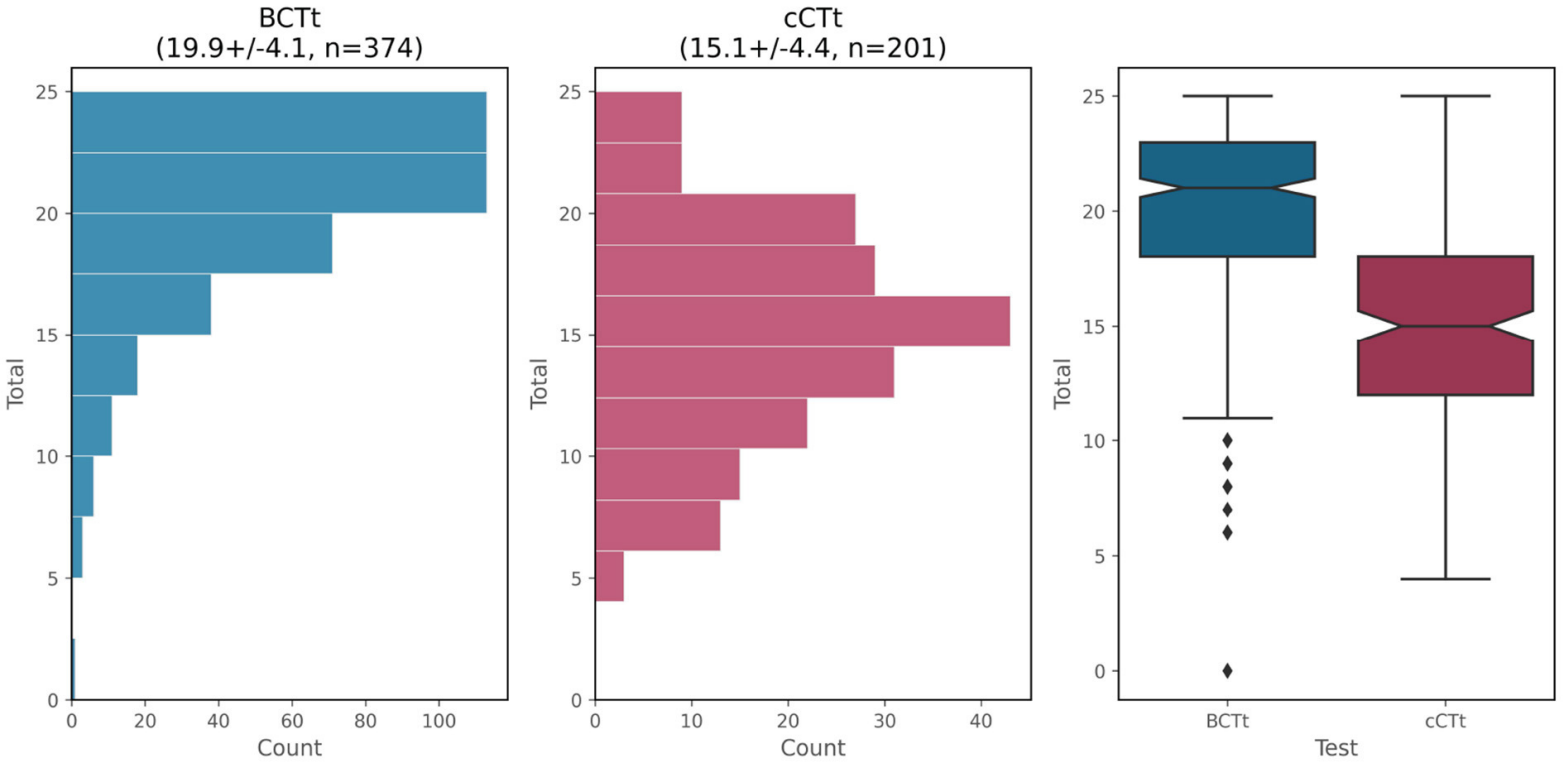
Score distribution for the BCTt and cCTt. The histogram and boxplots show the ceiling effect of the BCTt while the cCTt exhibits a normal distribution centered around 15/25 (i.e., 60%).

The distribution of scores obtained in the two tests (both out of a maximum of 25 points) is shown in Figure 3. The Shapiro-Wilk test of normality indicates that the distribution of the cCTt is normal (*p*>0.05 , fails to reject *H*_0_ ) and that the distribution of BCTt is not (*p* < 0.0001 , rejects *H*_0_ ). This is due to a ceiling effect, which is apparent for the BCTt (skew = −1.23 , kurtosis = 1.98 ), but is not present in the case of the cCTt (skew = −0.07 , kurtosis = −0.13 ).^4^ Neither instrument shows significant differences in scores between genders [one-way ANOVA *F*_*BCTt*_(1) = 0.19 , *p*_*BCTt*_ = 0.67 ; one-way ANOVA *F*_*cCTt*_(1) = 0.03 , *p*_*cCTt*_ = 0.86 ].

Where the BCTt is concerned, students in grade 4 (μ_4_ = 20.62 ± 3.66 ) perform significantly better than students in grade 3 (μ_3_ = 19.18 ± 4.16 ). Indeed, the one-way ANOVA indicates that the difference between grades is significant [*F*_(1)_ = 10.18 , *p* = 0.0016 , Δμ = 1.44 out of 25 ] with a medium-small effect size (Cohen's *d* = 0.37 ^5^ Lakens, 2013). This would appear to confirm the progression between grades on the BCTt observed in the original BCTt validation.

Where the cCTt is concerned, no significant differences exist between grades [one-way ANOVA *F*_(1)_ = 1.63 , *p* = 0.2 ]. The lack of distinction between grades in this sample is related to the fact that the grade 3 students are performing well on the test (μ = 14.64 ± 3.75 out of 25 ), and specifically as well as the grade 4 students (μ = 15.45 ± 4.68 ). Indeed, in the first study validating the cCTt, the grade 3 students scored an average of μ = 12.62 ± 5.18 (*n* = 711) and the grade 4 students μ = 15.49 ± 4.96 (*n* = 749) out of 25 .

### 3.2. Classical Test Theory

Cronbach's α (Bland and Altman, 1997) measurement of internal consistency of scales was used as an indicator of the instruments' reliability. According to the thresholds of Hinton et al. (2014) and Taherdoost (2016), both instruments exhibit high reliability (α_*BCTt*_ = 0.82>0.7 , α_*cCTt*_ = 0.78>0.7 ). Nonetheless, the individual item difficulties (i.e., the proportion of correct answers) and point biserial correlations (i.e., the difference between the high scorers and the low scorers of the sample population) provide useful insights into the developmental appropriateness of the instruments, by indicating which items could be revised to improve the validity of the instruments for the target populations.

**Figure 4:**
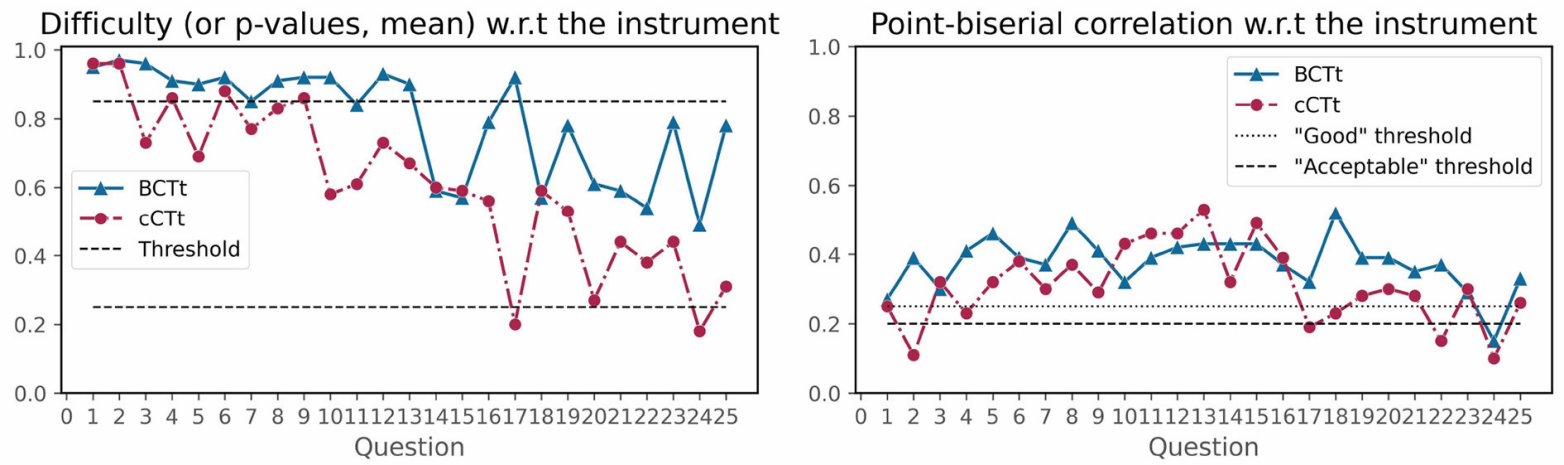
Classical Test Theory—Item Difficulty indices (i.e., the proportion of correct responses) on the left, and Point-Biserial Correlation on the right. Items with difficulty indices above the 0.85 threshold are considered too easy while items with difficulty indices below the 0.25 threshold are considered too difficult. Items with a point-biserial correlation above the 0.2 threshold are considered acceptable while those above 0.25 are considered good.

Figure 4 shows that both instruments present questions of decreasing difficulty (i.e., that are harder). The BCTt counts 13 questions which are above the maximum difficulty index threshold (i.e., are too easy) for the target age group, as opposed to 5 for the cCTt (including the 3 that were too easy in the original cCTt validation). The cCTt also exhibits two questions which are too hard (the same ones as in the original cCTt validation), which is not the case of the BCTt. Indeed, as Figure 4 shows, the BCTt covers a smaller range of item difficulties (BCTt difficulty indices min = 0.97 , max = 0.49 , range = 0.48 ; cCTt difficulty indices min = 0.96 , max = 0.18 , range = 0.79 ), lacking items in the lower half of the difficulty index range.

In terms of point-biserial correlation (see Figure 4), questions that could be revised for students in grades 3–4 are those below the 0.2 threshold. The metric indicates that only one item could be revised for the BCTt (question 24), while four items of the cCTt could be revised (questions 2, 17, 22, and 24). Interestingly, most of these questions were among the most difficult ones for the students.

Table 4 reports the Classical Test Theory analysis results for all questions in the two tests. Accounting for both difficulty indices and point biserial correlation, the number of questions that could be revised for students in grades 3 and 4 are higher for the BCTt (*n* = 14 ) than the cCTt (*n* = 8 ), as can be seen in Table 4.

**Table 4:** Full BCTt (Cronbach's α_*BCTt*_ = 0.82 ) and cCTt (Cronbach's α_*cCTt*_ = 0.78 ) Classical Test Theory Analysis.

			**BCTt**					**cCTt**			
**Q**	**Difficulty index**	**std**	**PBC**	**Drop alpha**	**Revision**	**Q**	**Difficulty index**	**std**	**PBC**	**Drop alpha**	**Revision**
1	**0.95**	0.22	0.27	0.82	x	1	**0.96**	0.19	0.25	0.78	x
2	**0.97**	**0.16**	0.39	0.82	x	2	**0.96**	0.19	**0.11**	0.78	x
3	**0.96**	0.2	0.3	0.82	x	3	0.73	0.44	0.32	0.77	
4	**0.91**	0.29	0.41	0.82	x	4	**0.86**	0.35	0.23	0.78	x
5	**0.9**	0.3	0.46	0.81	x	5	0.69	0.46	0.32	0.77	
6	**0.92**	0.27	0.39	0.82	x	6	**0.88**	0.32	0.38	0.77	x
7	**0.85**	0.35	0.37	0.82	x	7	0.77	0.42	0.3	0.77	
8	**0.91**	0.29	0.49	0.81	x	8	0.83	0.38	0.37	0.77	
9	**0.92**	0.27	0.41	0.82	x	9	**0.86**	0.35	0.29	0.78	x
10	**0.92**	0.27	0.32	0.82	x	10	0.58	0.49	0.43	0.77	
11	0.84	0.37	0.39	0.82		11	0.61	0.49	0.46	0.76	
12	**0.93**	0.25	0.42	0.82	x	12	0.73	0.45	0.46	0.77	
13	**0.9**	0.3	0.43	0.81	x	13	0.67	0.47	0.53	0.76	
14	0.59	0.49	0.43	0.81		14	0.6	0.49	0.32	0.77	
15	0.57	0.5	0.43	0.81		15	0.59	0.49	0.49	0.76	
16	0.79	0.41	0.37	0.82		16	0.56	0.5	0.39	0.77	
17	**0.92**	0.27	0.32	0.82	x	17	**0.2**	0.4	**0.19**	0.78	x
18	0.57	0.5	0.52	0.81		18	0.59	0.49	0.23	0.78	
19	0.78	0.42	0.39	0.82		19	0.53	0.5	0.28	0.78	
20	0.61	0.49	0.39	0.82		20	0.27	0.45	0.3	0.77	
21	0.59	0.49	0.35	0.82		21	0.44	0.5	0.28	0.78	
22	0.54	0.5	0.37	0.82		22	0.38	0.49	**0.15**	0.78	x
23	0.79	0.41	0.29	0.82		23	0.44	0.5	0.3	0.77	
24	0.49	0.5	**0.15**	0.83	x	24	**0.18**	0.38	**0.1**	0.78	x
25	0.78	0.41	0.33	0.82		25	0.31	0.46	0.26	0.78	

Q, question; Difficulty index, proportion of correct responses; std, standard deviation; PBC, Point-Biserial Correlation. Items that are too easy (i.e., μ>0.85 ), too difficult (i.e., μ < 0.25 ), or with a low point-biserial correlation (< 0.2 ) are marked in bold as elements which could be revised.

### 3.3. Item Response Theory (IRT)

#### 3.3.1. Verifying the unidimensionality to compare instruments through Confirmatory Factor Analysis

One criteria required to compare instruments through IRT is that the data measure the same latent trait. We thus employed Confirmatory Factor Analysis (CFA) as done by Kong and Lai (2022), with a Diagonally Weighted Least Squares estimator to account for the binary inputs (see Table 5 for the fit indices). The Kaiser, Meyer, Olkin (KMO) measure of sampling adequacy indicates that the data is appropriate for factor analysis in both cases. Bartlett's test of sphericity also suggests that there is sufficient significant correlation in the data for factor analysis. For the full instruments (with 25 items) the model fit indices are also adequate in terms of the χ^2^ criteria statistic, the CFI and TLI indices for both instruments. The RMSEA is below 0.6 in both cases. Finally, the SRMR is considered acceptable for the cCTt and just shy of the limit for the BCTt (*SRMR*_*BCTt*_ = 0.084 ). The modification indices for the BCTt-CFA indicate high correlations between 3 items from the BCTt (Q14, Q15, and Q18) which address the notions of complex loops. Removing item 15 from the factor analysis improves the model fit and meets the threshold requirements for the different fit indices (see Table 5). Furthermore, we exclude items with low CFA factor loadings (< 0.2 ) from the IRT analysis. Please note that all remaining items have significant factor loadings and that the excluded items correspond to questions which have low point biserial correlations (namely Q24 in the BCTt, and Q2, Q17, Q22, and Q24 in the cCTt). The corresponding fit indices for the final 1 factor CFA are provided in Table 5. With these adjustments, a 1 factor structure appears suitable for both instruments (when excluding Q15 and Q24 from the BCTt, and Q2, Q17, Q22, and Q24 from the cCTt).

**Table 5:** Confirmatory factor analysis fit indices for unidimensionality.

	**Modification**	**KMO**	**Bartlett's test of sphericity**	**χ^2^**	**χ^2^/df**	**CFI**	**TLI**	**RMSEA**	**SRMR**
BCTt (25 items)		0.84	χ^2^(300) = 2026, *p* < 0.001	χ^2^(275) = 464, *p* < 0.001	True	0.92	0.913	0.43, 90*%ci* = [0.036 − 0.05]	0.084
BCTt (24 items)	Removing Q15 (high correlations with Q14 % Q18)	0.84	χ^2^(276) = 1825, *p* < 0.001	χ^2^(252) = 357, *p* < 0.001	True	0.945	0.940	0.033, 90*%ci* = [0.025 − 0.041]	0.076
BCTt (23 items)	Removing Q24 (low factor loading)	0.84	χ^2^(253) = 1779, *p* < .001	χ^2^(230) = 322, *p* < 0.001	True	0.951	0.946	0.033, 90*%ci* = [0.024 − 0.041]	0.074
cCTt (25 items)		0.75	χ^2^(300) = 877, *p* < 0.001	χ^2^(275) = 350, *p* = 0.001	True	0.935	0.929	0.037, 90*%ci* = [0.024 − 0.049]	0.077
cCTt (21 items)	Removing Q2, Q17, Q22, Q24 (low factor loading)	0.77	χ^2^(210) = 761, *p* < .001	χ^2^(189) = 216, *p* < 0.089	True	0.975	0.972	0.027, 90*%ci* = [0.000 − 0.043]	0.071

#### 3.3.2. Comparing the instruments

As indicated previously, we only consider the 1-PL and 2-PL models in our study due to the low sample sizes which prevent us from finding stable solutions in the case of the 3-PL model and prevent us from converging in the case of the 4-PL model (see global model fit indices for the 1-PL and 2-PL models in Table 6). For both the BCTt and the cCTt, the 2-PL model was selected as an ANOVA indicated that the 2-PL model improved the fit significantly compared to the 1-PL model in both cases [$\chi_{BCTt}^{2}\left(22\right)=62.92$, *p*_*BCTt*_ < 0.0001, $\chi_{cCTt}^{2}\left(20\right)=79.84$, *p*_*cCTt*_ < 0.0001]. Individual item discrimination, difficulties, and fit indices are provided for the 2-PL models in Table 7. The results indicate that the χ^2^/*df* < 3 criterion is achieved for all items, and that all but three items have RMSEA just shy of the 0.6 threshold (considering that the rounded values would be equal to 0.6 these can be considered acceptable, Ockey and Choi, 2015). We then verify the local independence using Yen (1984)'s Q3 statistic and find that it is below the 0.3 threshold for all pairs of items in the BCTt and in the cCTt.

**Table 6:** IRT model parameter fit indices for 1-PL and 2-PL models with the BCTt and cCTt.

		***M*_2_**	***df***	***p***	**RMSEA**	**ci RMSEA 5%**	**ci RMSEA 95%**	**SRMR**	**TLI**	**CFI**
BCTt (23 items)	1-PL	514	253	0.000	0.053	0.046	0.059	0.098	0.929	0.929
	2-PL	415	230	0.000	0.046	0.039	0.053	0.068	0.945	0.950
cCTt (21 items)	1-PL	392	210	0.000	0.067	0.056	0.077	0.102	0.849	0.849
	2-PL	294	189	0.000	0.053	0.041	0.065	0.075	0.903	0.913

**Table 7:** BCTt and cCTt item parameters and fit indices.

				**BCTt**								**cCTt**			
**Item**	**Dscr**	**Dffc**	**S-χ^2^**	**df S-χ^2^**	**RMSEA**	**p S-χ^2^**	**S-χ^2^/df**	**Item**	**Dscr**	**Dffc**	**S-χ^2^**	**df S-χ^2^**	**RMSEA**	**p S-χ^2^**	**S-χ^2^/df**
					**S-χ^2^**								**S-χ^2^**		
Q1	1.29	-2.8	11.5	8	0.034	0.175	1.44	Q1	2	-2.38	3.3	2	0.058	0.191	1.66
Q2	2.79	-2.28	2.3	2	0.019	0.323	1.13	Q3	0.95	-1.27	13.7	11	0.035	0.251	1.24
Q3	1.73	-2.47	3.2	6	0.0	0.786	0.53	Q4	0.76	-2.6	15.2	10	0.052	0.124	1.52
Q4	1.82	-1.85	13.8	11	0.026	0.245	1.25	Q5	0.95	-1	11.6	11	0.017	0.392	1.06
Q5	2.41	-1.6	20.3	9	0.058	0.016	2.26	Q6	2.01	-1.56	9	7	0.038	0.254	1.28
Q6	1.66	-2.03	13.1	11	0.022	0.29	1.19	Q7	1.02	-1.4	11	11	0.0	0.447	1
Q7	1.38	-1.67	15.8	12	0.029	0.2	1.32	Q8	1.46	-1.45	9.5	9	0.016	0.396	1.05
Q8	2.56	-1.6	11.8	9	0.029	0.224	1.31	Q9	1.47	-1.67	16.2	9	0.064	0.062	1.8
Q9	2.16	-1.82	8.6	10	0.0	0.574	0.86	Q10	1.52	-0.33	14.1	10	0.046	0.167	1.41
Q10	1.41	-2.26	18.2	11	0.042	0.077	1.66	Q11	1.76	-0.4	5.3	10	0.0	0.871	0.53
Q11	1.42	-1.55	14.6	13	0.018	0.334	1.12	Q12	1.98	-0.82	14.3	8	0.063	0.075	1.79
Q12	2.02	-1.95	9.9	10	0.0	0.451	0.99	Q13	2.75	-0.53	7.9	7	0.026	0.338	1.13
Q13	1.93	-1.73	13.3	11	0.024	0.275	1.21	Q14	0.98	-0.49	17.1	10	0.061	0.071	1.71
Q14	1.31	-0.36	14.7	10	0.035	0.145	1.47	Q15	2.35	-0.3	13	8	0.057	0.112	1.62
Q16	1.25	-1.37	19.3	13	0.036	0.113	1.49	Q16	1.09	-0.27	5.7	10	0.0	0.837	0.57
Q17	1.43	-2.22	11.8	11	0.014	0.383	1.07	Q18	0.53	-0.75	16.3	12	0.043	0.178	1.36
Q18	1.77	-0.26	8.6	8	0.014	0.379	1.07	Q19	0.62	-0.22	9.2	12	0.0	0.687	0.77
Q19	1.21	-1.3	11.4	11	0.01	0.408	1.04	Q20	0.65	1.66	12	10	0.032	0.284	1.2
Q20	1.13	-0.5	9.2	11	0.0	0.607	0.83	Q21	0.49	0.53	13.4	12	0.024	0.343	1.11
Q21	0.85	-0.52	7.8	11	0.0	0.733	0.71	Q23	0.63	0.38	9.5	11	0.0	0.577	0.86
Q22	1.08	-0.18	17	10	0.043	0.073	1.7	Q25	0.5	1.66	11.3	11	0.011	0.421	1.02
Q23	0.86	-1.74	18	13	0.032	0.157	1.39								
Q25	0.92	-1.63	14.2	13	0.016	0.357	1.1								

Dscr, Discrimination; Dffc, Difficulty.

**Figure 5:**
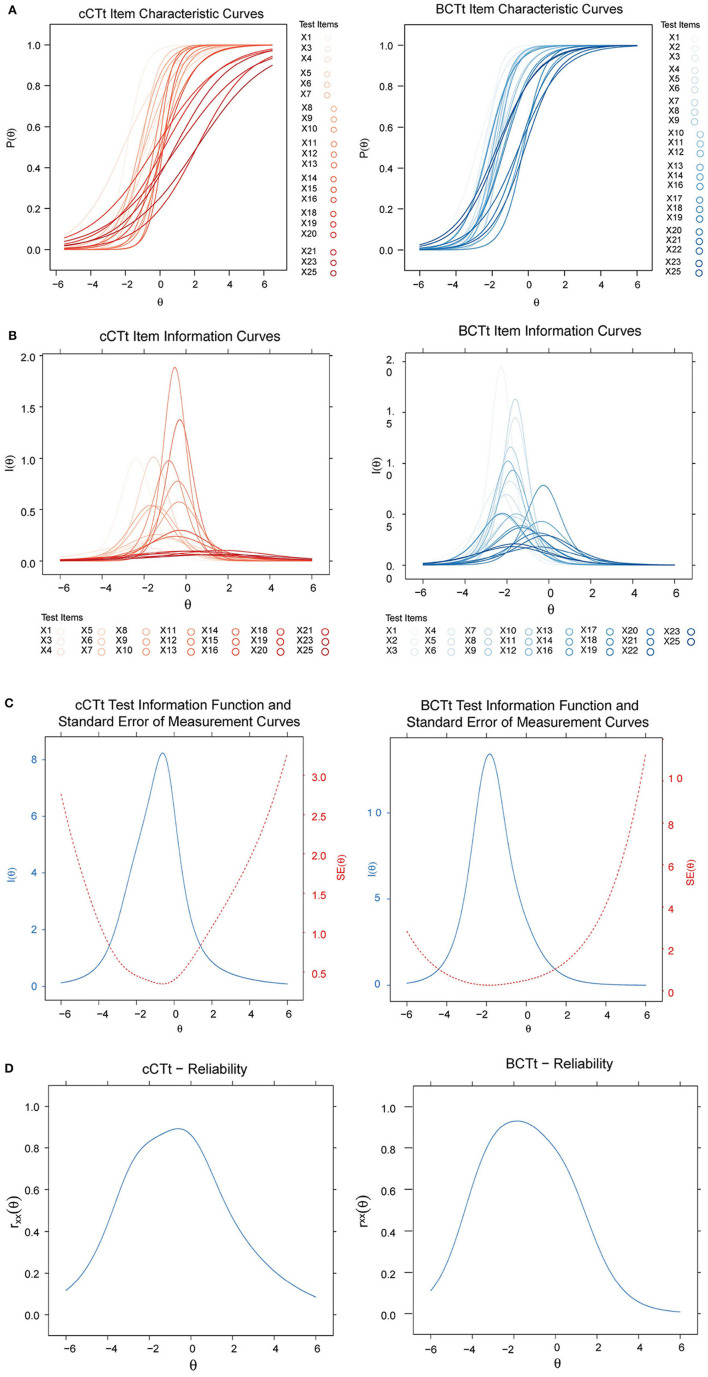
Item Response Theory curves for the BCTt and the cCTt. **(A)** Item Characteristic Curves (ICC). The figure shows that the items have varying difficulties and discrimination (slopes), with BCTt items showing higher discriminability in the low ability range and cCTt items showing higher discriminability in the low and medium ability ranges. **(B)** Item Response Theory Item Information Curves (IIC). Items in both instruments provide varying amount of information at different ability levels. Similarly to the ICC curves in Panel **(A)**, the information of the BCTt is mainly in the low ability range, while the information of the cCTt is in the low and medium ability ranges. Item Response Theory curves for the BCTt and the cCTt. **(C)** Test information function (TIF). The TIF being the sum of each instruments' Item Information Curves [see Panel **(B)**], the results confirm prior observations: the BCTt provides most of its information in the low ability range while the cCTt provides most information in the low and medium ability ranges. **(D)** Reliability at different ability levels. The figures show that both instruments have low reliability in the high ability range. The BCTt reliability peak is shifted toward the lower ability range while the cCTt reliability peak is toward the medium ability range. Please note that the marginal reliability *r*_*xx*_ for the BCTt is *r*_*xx*_(*BCTt*) = 0.75, and for the cCTt *r*_*xx*_(*cCTt*) = 0.80.

**Figure 6:**
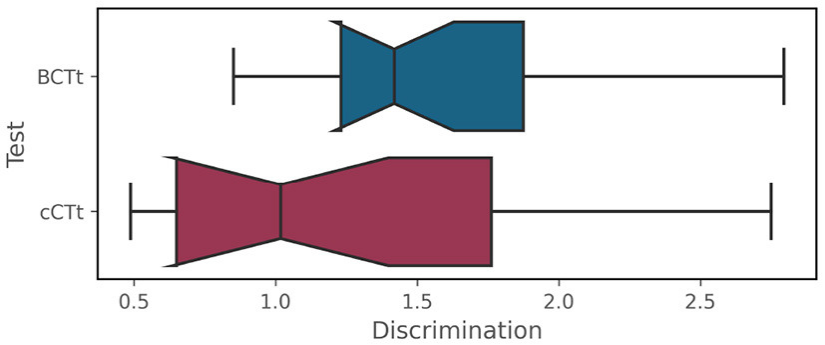
Item Response Theory BCTt–cCTt item discrimination comparison [one-way ANOVA *F*_(1)_ = 3.11 , *p* = 0.085 ].

The results of the IRT analyses are shown in Figures 5A–D. While the Item Characteristic curves (Figure 5A) appear to indicate that the BCTt questions have higher “discrimination power” than the cCTt questions, this difference is not significant [one-way ANOVA *F*_(1)_ = 3.11 , *p* = 0.085 , see Figure 6]. This means that both tests are as good at discriminating between students, however where they discriminate best differs^6^. The Item Information Curves (Figure 5B) shows that the BCTt questions provide most information in the low ability range, while the Item Information is more distributed along the low-medium range for the cCTt. The resulting TIFs (Figure 5C) therefore confirm that the BCTt is better at discriminating between students with low ability, while the cCTt is better at discriminating between low-medium abilities. As such, the IRT findings support that the cCTt overall fits grade 3–4 individuals and it decently works all along the ability range.

### 3.4. Limitations

As in all studies, the study presents certain limitations. Aside the inherent limitations pertaining to the specific use of Classical Test Theory and Item Response Theory which are well documented in the literature, the following elements are specific to the current study.

The instruments were tested on two populations from different schools, one year apart, and may thus differ in their CT abilities. While the students in the same grades should be expected to have the same level of CT-skills, this may not be the case. However, certain elements help mitigate this risk and counter the limitation: the schools are in the same country and district and thus follow the same mandatory curriculum (which does not include CS or CT), the measurements took place at the same time of the academic year, and we employed IRT as it tends to be sample agnostic.

The relatively small sample sizes prevented us from testing more complex models, such as 3-PL and 4-PL models. Indeed, larger sample sizes, in particular for the cCTt (*n* = 200), would have likely improved the model fit and reliability of the item difficulty and discrimination indices. These indices should only be considered as indicative of where the test provides more information, also since the IRT analysis was conducted on a subset of the items to meet the unidimensionality criteria. However, please note that the IRT analysis was also conducted with the full subset of items (although not presented in the article) and lead to the same conclusions. Such an analysis is possible as the violation of the unidimentionality criteria leads to “an overestimation of the discrimination parameter, (ii) with little impact on the difficulty estimation” (Kahraman, 2013; Rajlic, 2019), with “the impact on the estimated parameters [being] smaller the closer we are to the unidimensionality criteria” (Kahraman, 2013; Rajlic, 2019). Given the small samples and the fact that the IRT parameters were estimated on a subset of the items, it would be best to avoid using the IRT parameter estimates of the present study, in particular for the cCTt, to estimate the students' abilities on the latent ability scale.

## 4. Recommendations for the use of the BCTt and the cCTt

Considering (i) the present BCTt-cCTt comparison, (ii) the results of the BCTt validation conducted by Zapata-Cáceres et al. (2020) over grades 1–6, and (iii) the cCTt validation conducted by El-Hamamsy et al. (2022a) over grades 3–4, we propose the following recommendations with respect to these two instruments for grades 3–4:

- The cCTt should be preferred for grades 3–4 as it differentiates better between students in this age group and ability level, in addition to discriminating moderately well along the entire ability range. The cCTt is thus better suited to evaluate the efficacy of the intervention itself, in a pre- post-test design.
- The BCTt could be employed for low-ability students in grades 3–4, depending on the assessors' prior knowledge of the context and the students being assessed given the good discriminability the BCTt offers in grades 3–4 for low ability students.
- The BCTt could be employed as a screening mechanism to identify low-ability students which could prove useful for practitioners prior to an intervention, e.g., to ensure that the intervention is well-tailored to the abilities of the students and ensure that nobody is “left behind.”

## 5. Discussion and conclusion

The BCTt and the cCTt are two instruments that expand the portfolio of validated CT assessments, in particular, at the level of primary education. These instruments overlap in their target age ranges, notably in grades 3–4, and had not yet been compared psychometrically for those age groups. This study thus looked to establish the limits of validity of the two instruments by providing a detailed comparison of their psychometric properties on data acquired from 575 students (374 doing the BCTt and 201 doing the cCTt). Indeed, as:

1. The BCTt and the cCTt were validated in different countries, and thus potentially different contexts
2. There were only *n* = 52 grade 4 students in the BCTt validation, and *n* = 0 grade 3 students, with limited psychometric analyses conducted for the BCTt in those grades specifically.

The present study looked to conduct a detailed psychometric analysis of the BCTt in grades 3–4 (which was not yet conducted) and compare the validity of the two instruments on a large and comparable pool of grade 3–4 students from a third, and single, country.

The findings from the psychometric analyses of the two instruments help re-establish their validity in grades 3 and 4 with both a new population and with students from a new country (here *n* = 575in Portugal, while the cCTt was validated with *n* = 1,519 grade 3–4 students in Switzerland, El-Hamamsy et al., 2022a, and the BCTt with *n* = 299 grade 1–6 students in Spain, Zapata-Cáceres et al., 2020). Where the cCTt is concerned, while there were no differences between students in grades 3–4 in the present sample, the general conclusions drawn from the Classical Test Theory analysis and overall IRT are coherent with those obtained by El-Hamamsy et al. (2022a). Where the BCTt is concerned, the results confirm the ceiling effect observed in grade 4 in the original study (Zapata-Cáceres et al., 2020) and extend it to students in grade 3 who were not part of the initial pool of students who were administered the BCTt. The psychometric comparison indicates that *the cCTt should be preferred for students in grades 3 and 4*, as students already have a good assimilation of basic CT concepts pertaining to sequences and loops. Therefore, students in grades 3–4 perform too well on the easier BCTt (which employs smaller 3 × 3 grids), giving rise to a ceiling effect. The *BCTt should instead be preferred if the objective is to discriminate between students with low abilities in grades 3 and 4*.

The findings are consistent with other studies that found that simple loops are already mastered in early primary school (Montuori et al., 2022), with very young students (starting 3 years old) already being able to solve algorithmic problems and their results improving with age (Piatti et al., 2022). As CT skills relate to students' numerical, verbal, and non-verbal reasoning abilities (Tsarava et al., 2022), it is likely that the findings align with students' maturation, increase in working memory (which is required to achieve tasks, Cowan, 2016), and executive functions over time. Therefore, as students get older, they should be able to deal with more complex computational concepts (e.g., conditionals and while loops), including those with more complex perceptual configurations (e.g., the 4 × 4 grids), corroborating the differences observed between both instruments. Future work should therefore consider continuing to refine the limits of validity of the instruments. Indeed, refinement studies are common in educational psychology, with similar work having already been undertaken for (i) the original CTt (aimed at 10–16 year old students) to improve it's validity for 16 year old students and above (Guggemos et al., 2022), and (ii) The TechCheck and it's variants to improve the validity for kindergarden students (Relkin et al., 2020; Relkin and Bers, 2021).

Two key takeaways emerge from the present study:

1. The importance of building and validating CT assessments for each specific age: children in the early stages of education undergo rapid cognitive development, so an instrument designed for a specific age range is likely to be too difficult for those immediately younger and too easy for those immediately older.
2. The importance of psychometrically comparing existing, overlapping CT instruments to establish their limits of validity. By providing detailed comparisons, researchers and practitioners may be able to choose the assessment in an informed way, and in accordance with their requirements and objectives.

As numerous researchers have put forward, instruments such as the BCTt and the cCTt should be combined with other forms of assessments in a systems of assessments (Grover et al., 2015; Román-González et al., 2019; Weintrop et al., 2021a) to accurately measure the full range of competencies at play when considering CT (Brennan and Resnick, 2012; Piatti et al., 2022). The systems of assessments could therefore include other instruments which assess CT practices such as the test by Li et al. (2021), employ direct observations of students' thought processes and strategies (Lye and Koh, 2014; Chevalier et al., 2020), or learning analytics and educational data mining techniques (Cock et al., 2021; Nasir et al., 2021; Zapata-Cáceres and Martín-Barroso, 2021). Complementary assessments would not only help gain a more accurate and in-depth picture of student learning but also feed into the learning activity design and intervention process (Chevalier et al., 2022). For completeness, the system of assessments should also include instruments that measure CT perspectives (e.g., such as those developed for high school, Yagci, 2019 and undergraduates, Korkmaz et al., 2017).

Provided that validation is a multi-step process that requires “collect[ing] multiple sources of evidence to support the proposed interpretation and use of assessment result[s] [and] multiple methodologies, sources of data, and types of analysis” (Gane et al., 2021), it is important to note that the BCTt and cCTt may still undergo further validation by including evidence of criterion validity. This can be achieved through several means. The first is comparing with other existing validated assessments. For instance, Relkin et al. (2020) compared the TechCheck with the TACTIC-KIBO, while (Li et al., 2021) went one step further and correlated the CTA-CES with reasoning, spatial abilities, and verbal abilities. The second is establishing the test's predictive validity, for example by establishing whether the instrument can predict academic performance and coding achievement as done by Román-González et al. (2018). The third is determining the instruments' concurrent validity, that is to say seeing whether the instrument is able to distinguish between two groups that differ, for instance novices and experts, or according to students expressed digital proficiency as done by Li et al. (2021).

## Data availability statement

The data presented in this study can be found on Zenodo (El-Hamamsy et al., 2022b).

## Ethics statement

The studies involving human participants were reviewed and approved by Comité de Ética de la Investigación de la Universidad Rey Juan Carlos. Written informed consent to participate in this study was provided by the participants' legal guardian/next of kin.

## Author contributions

LEH, MZC, PM, BB, and EMB: conceptualization. LEH, MZC, and PM: methodology. MRG: validation. LEH: formal analysis, writing—original draft and preparation, and visualization. PM: investigation. PM and LEH: data curation. LEH, MZC, PM, JD, BB, EMB, and MRG: writing–review and editing. BB and EMB: supervision. All authors contributed to the article and approved the submitted version.

## Footnotes

^1^Please note that the full BCTt is available upon request to the co-authors of Zapata-Cáceres et al. (2020), and the cCTt items are presented in El-Hamamsy et al. (2022a) with an editable version available upon request to the co-authors of the article.

^2^In the original validation of the BCTt by Zapata-Cáceres et al. (2020) they obtained average scores of μ_*grade*1_ = 16.52 ± 3.31 , μ_*grade*2_ = 16.78 ± 2.49 , μ_*grade*4_ = 21.57 ± 3.04 , μ_*grade*5_ = 21.84 ± 2.61 , μ_*grade*6_ = 21.72 ± 2.62 out of 25. Please note that their sample did not include grade 3 students.

^3^The data is available on Zenodo (El-Hamamsy et al., 2022b).

^4^Skew (i.e., the asymmetry of a distribution) and kurtosis (i.e., the location of the peak of a distribution) of a normal distribution are close to 0 (Kim, 2013).

^5^Cohen's *d* effect size is a quantitative measure of the magnitude of the observed difference. It is a standardized measure of the difference between the two means which is calculated by dividing the difference of the means by the standard deviation. Cohen suggested that 0.2 is a small effect size, 0.5 a medium effect size, and 0.8 a large effect size (Lakens, 2013).

^6^The “discrimination power” of the instrument which relates to how high the discrimination is over all the questions of the assessment and is provided by slope of the ICCs, maximum values of the IICs. This is related to where the assessment, and thus the individual questions, discriminate best (which is provided by the *y* = 0.5 crossing of the ICCs, or the peak of the IICs).
